# More micrometastases, more recurrence? The role of qPCR of PSA mRNA in lymph nodes during prostatectomy

**DOI:** 10.1007/s00345-024-05414-2

**Published:** 2025-01-05

**Authors:** Johannes Troidl, Alexander Fehr, Burkhard Jandrig, Jens Köllermann, Anke Lux, Daniel Baumunk, Melis Gür, Uwe-B. Liehr, Markus Porsch, Johann J. Wendler, Simon Blaschke, Martin Schostak

**Affiliations:** 1 Department of Urology, Urooncology, Robot-assisted and Focal Therapy, University Hospital Magdeburg, Otto-von Guericke University Magdeburg, Leipziger Str. 44, 39120 Magdeburg, Germany; 2 Senckenbergische Institute für Pathology & Genetics, Frankfurt/Main, Germany; 3 Institute of Biometry and Medical Informatics, University Hospital, Otto-Von Guericke University, Magdeburg, Germany; 4 Praxis Baumunk & Baumunk, Backnang, Germany; 5 Urologen am Hassel, Magdeburg, Germany

**Keywords:** Biochemical recurrence, Lymph node metastases, Prostate cancer, QPCR, Radical prostatectomy

## Abstract

**Background and objectives:**

Radical prostatectomy is a standard treatment for prostate cancer, yet about 30% of patients experience rising biochemical markers within a decade post-surgery. Pelvic lymph node sampling during prostatectomy assesses potential lymph node metastases, but standard histological assessments, which typically examine only 2–3 tissue sections, often miss occult metastases. This study assesses the effectiveness of qPCR in detecting PSA coding KLK3 mRNA for identifying lymph node metastases post-prostatectomy and explores the correlation between PSA-mRNA and biochemical recurrence.

**Methods:**

A cohort of 157 patients who underwent radical prostatectomy with lymphadenectomy were examine. On average, 24.7 lymph nodes were removed per patient. Among them, 108 patients reached PSA value below 0.1 ng/ml without receiving additional therapy, and 106 were followed up over a duration of 5.4 years. This subgroup is of particular interest because it allows for the investigation of the correlation between the occurrence of PSA-mRNA in lymph nodes and later biochemical recurrence.

Key findings and limitations

qPCR of PSA-mRNA identified 47 out of 108 positive cases (43.5%), while histopathological examination only detected 16 out of 108 cases (14.8%). From the followed-up subgroup 37 out of 106 patients (34.9%) experienced biochemical recurrence. It is noteworthy that qPCR yields more positive findings, regardless of the presence of biochemical recurrence.

**Conclusion and clinical implications:**

The study findings illustrate that qPCR consistently outperforms conventional histology in detecting lymph node metastases, regardless of biochemical recurrence. The hypothesis that qPCR is better at predicting later biochemical recurrence than conventional histology has not been confirmed.

**Supplementary Information:**

The online version contains supplementary material available at 10.1007/s00345-024-05414-2.

## Introduction

Metastatic tumors, which spread from their primary site, are a major cause of death for many cancer patients. These tumors occur when cells from the primary tumor migrate to other parts of the body. Notably, prostate cancer has become increasingly significant as the most common cancer among men and the second leading cause of cancer-related death in developed nations [2, 4].

Radical prostatectomy is a well-established treatment for prostate cancer. However, about 35% of patient experience a rising PSA within 10 years after surgery, indicating a return of cancerous cells [11]. While low grade tumors tend to remain free of biochemical recurrence for an extended period after the procedure, patients with more advanced prostate cancer have a higher risk of disease progression [12].

Patients with lymph node metastases benefit less from local therapies such as adjuvant or salvage radiotherapy; therefore, an early diagnosis could render these therapies unnecessary. Nowadays, metastatic disease is intended to be treated with systemic therapy (EAU Guidelines Office, o. J.). It is conceivable that an earlier initiation of treatment may have a favorable prognostic impact.

Standard methods of assessing lymph nodes, like conventional histology, offer limited insights because the tissue is typically divided into only a few sections. Consequently, metastases located between these sections often remain undetected. Köllermann et al. demonstrated decades ago that analyzing around thousands of sections of a single lymph node would be necessary to reliably detect or rule out metastasis [5].

PSA expression, found exclusively in prostate cancer cells and usually not detectable in lymph node tissue, serves as a highly sensitive marker. This characteristic was first described by Ablin et al. in 1976 [1]. In our research, we employ qPCR to investigate the presence of PSA mRNA in lymph nodes. A positive result indicates the existence of prostate cancer cells, suggesting the onset or established presence of lymph node metastases, including micrometastases.

**Fig. 1:**
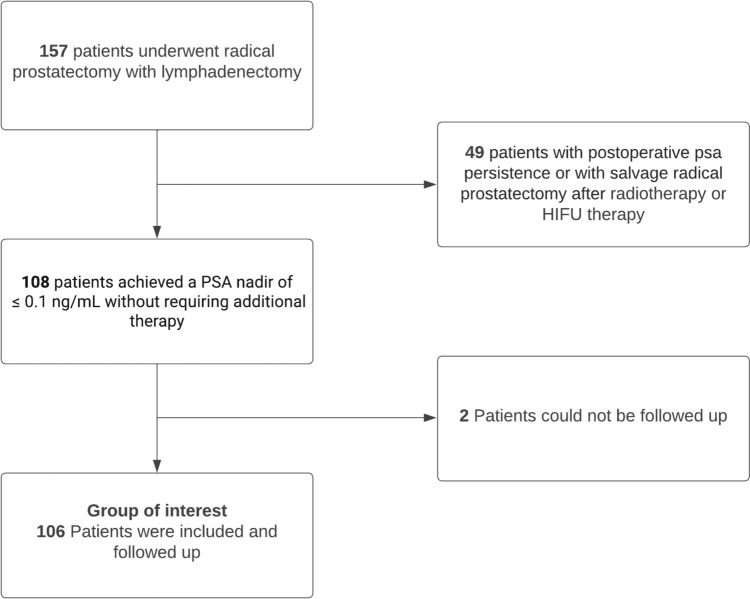
Flow Chart. This figure illustrates the number of patients included in this study

The first aim of this study was to compare the sensitivity of these findings with conventional histology. Then, as a second objective, we investigated the practical significance by following up patients 5.4 years after surgery for any biochemical recurrence. In this study, we adhere to the established definition of biochemical recurrence as outlined in the German guidelines: an increase in PSA from undetectable levels to above 0.2 ng/mL in at least two tests, or a single increase above 0.4 ng/mL (Fig. 1).

### Patients and methods

This prospective study enrolled a total of 157 patients with prostate cancer, who underwent either retropubic or robot-assisted radical prostatectomy with extended pelvic lymphadenectomy. The investigation was carried out at the Department of Urology, Urooncology, Robot-assisted and Focal Therapy at Magdeburg University Hospital during the period between 11/2013 and 03/2021.

All patients signed a written informed consent approved by the Medical Ethics Committee of the Otto-von-Guericke University Magdeburg (# 87/11). Analysis was carried out in accordance with the International Conference on Harmonisation (ICH)-approved Good Clinical Practice (GCP) and Good Laboratory Practice (GLP) guidelines and regulation.

The lymphadenectomy procedure encompassed the bilateral removal of pelvic lymph nodes from the obturatoric fossa as well as from the drainage regions of the external and internal iliac vessels. Subsequent to resection, the lymph nodes were subjected to manual inspection, dissection, and division under aseptic separate conditions preventing contamination.

One half of the extracted pelvic lymph node samples was sent to the Institute of Pathology at University Hospital Magdeburg for standard histological examination. Histopathological evaluation of the samples conformed to the principles outlined by the WHO. After bisecting the lymph nodes along the longitudinal axis, the portion of lymph node sent to pathology department was fixed in 4% formalin overnight and further processed according to the standard tissue protocol. After paraffin embedding, the histopathologic workup of the lymph node tissue was based on the analysis of 1–2 hematoxylin–eosin (H&E) stained sections per lymph node, derived from one level of the paraffin-embedded tissue.

The prostatectomy specimens were inked entirely on their surfaces and processed according to the Stanford protocol using serial transverse sections at 3 mm [6].

The remaining half of the tissue specimens was immediately shock-frozen in liquid nitrogen and stored at a temperature of − 80 °C.

The lymph node packages were stored separately based on their collection site. There were six categories of the extended pelvic LAE: left and right obturatorial region, Iliaca interna left and right, and Iliaca externa left and right. The analysis was conducted equivalent for each specific tissue area.

Total RNA was isolated using the RNeasy Plus Universal Mini Kit (Qiagen, Hilden, Germany) in accordance with the provided guidelines from the manufacturer.

cDNA was synthesized from 1 µg total RNA using the High Capacity cDNA Reverse Transcription kit according to the recommendations of the manufacturer (Applied Biosystems, Foster City, CA). QPCR was performed using the TaqMan Universal PCR Master Mix kit on an ABI StepOnePlus^™^ Real-Time PCR System (Applied Biosystems, Foster City, CA, USA). Primers for two different FAM labeled KLK3 (encoding kallikrein related peptidase 3, PSA) assays (ID: Hs02576345_m1 and Hs03063374_m1) were obtained from the Gene Expression Assay collection (Thermo Fisher, Darmstadt, Germany). Thermal cycling conditions comprised an initial denaturation step at 95 °C for 10 min and 40 cycles at 95 °C for 15 s and 60 °C for 1 min. We also quantified transcripts of the human ACTB gene coding for ß-actin as well as GAPDH as endogenous RNA controls using VIC-MGB labeled Pre-Developed Assay Reagents (Applied Biosystems, Foster City, CA, USA). The analysis was facilitated by StepOne Software version 2.1 (Applied Biosystems, Darmstadt, Germany). Exogenous positive controls were comprised of cDNA solutions from the cell lines LNCaP (ATCC #CRL-1740™) and 22RV1 (ATCC # CRL-2505^™^). Experiments were performed with triplicates and in duplicate.

The data of the histopathological analysis of the prostatectomy specimens were obtained from the Institute of Pathology at Magdeburg University Hospital.

**Fig. 2:**
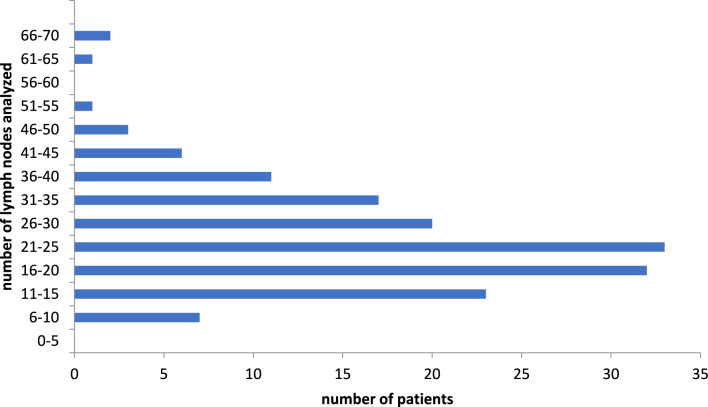
Number of lymph nodes analyzed from 157 patients. This graph illustrates the total number of lymph nodes that were subjected to histological analysis in the study cohort. In all, 3882 LNs were analyzed; y-axis shows the number of cases and the x-axis the number of LNs per patient

**Fig. 3:**
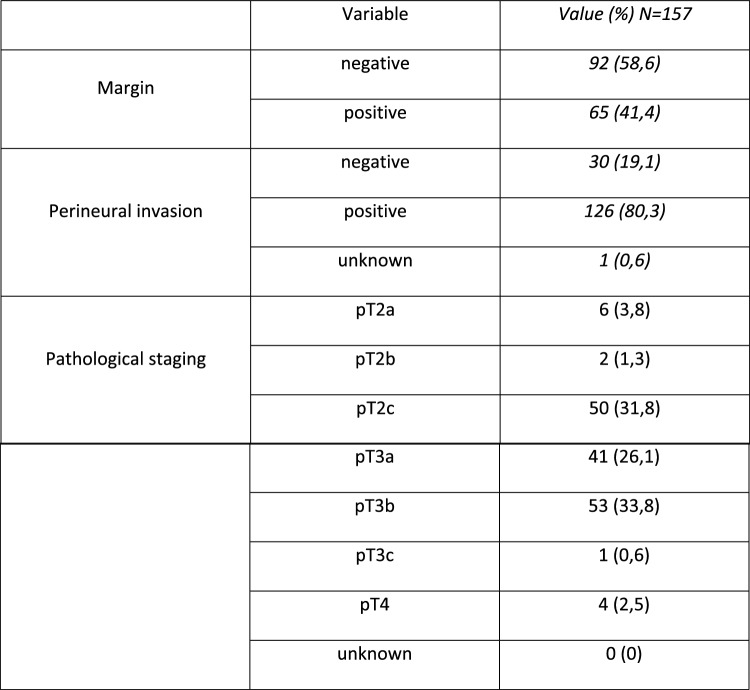
Additional Data. This graph presents supplementary data related to the study parameters, providing further insights into the research findings

Patient follow-up was performed by telephone surveys by the treating urologists. In addition to the latest PSA value and PSA nadir, information data were pooled whether and when the patient achieved a PSA value below 0.1 ng/ml. Moreover, the possible time and the cause of death were determined. The patients were followed up for an average of 5.4 years, with a standard error of 0.2 years (Figs. 2, 3).

## Results

The cohort comprised 108 patients who achieved a PSA nadir of ≤ 0.1 ng/mL after prostatectomy without requiring additional therapy, such as adjuvant radiotherapy. Salvage operations following radiotherapy or HIFU therapies were also excluded. The lymph nodes were examined both histologically and in the laboratory with an average of 24.7 (SD: 11) lymph nodes and 3,882 in total.

Out of the total of 108, 106 patients revealed a complete follow-up of at least 2 years. Out of these 106 patients, 37 (34.9%) had a biochemical recurrence disease.

Regarding the first objective, comparing the sensitivity of qPCR and conventional histology, it is noteworthy that the conventional histopathologic group detected malignant foci of lymph nodes in 14.8%. In contrast, qPCR identified PSA positive lymph nodes in 43.5% of patients.

Considering these data, it can be stated that qPCR is significantly more sensitive than conventional histology in detecting lymph node (micro)metastases.

When analysing the second objective of this study if the additionally detected patients with occult metastases in lymph nodes were more likely to suffer biochemical recurrence it is evident that qPCR has a lower positive predictive value than histology (40.4% qPCR vs. 50.0% histology), and a similar negative predictive value (69.5% qPCR vs. 67.8% histology).

## Discussion

Nowadays, patients with prostate cancer and metastases are subjected to much more intensive pharmacological and local treatment. In recent years, more sensitive metastatic detection methods such as PSMA-PET/CT have been implemented and the number of combinations of therapies has increased, culminating in the so-called triplet, a combination of androgen deprivation therapy (ADT), new hormone therapy (NHT), and docetaxel chemotherapy. Conversely, since the publication of the RADICALS-RT trial, adjuvant radiotherapy, once an almost automatic adjunct to surgery, is no longer recommended and is only considered in the highest risk cases, such as GLEASON 10 with established positive surgical margin [10].

Based on the hypothesis that pre-existing micrometastases in lymph nodes during surgery will likely lead to a faster systemic spread, we initiated this elaborate molecular project. Our expectations encompassed not only an improved sensitivity for metastases using qPCR compared to conventional histology but also a higher likelihood of positive results in patients with biochemical recurrence. Given that a systemic recurrence carries greater prognostic significance than a pure local recurrence, it was also our intention to spare the lymph node-positive group from the previously widespread but often unnecessary radiation of the prostatic fossa. In principle, the removal of prostate cancer-affected lymph nodes can potentially lead to a cure if micrometastasis is confined to this specific region. Messing et al. demonstrated in a 12-year follow-up study that 50% of patients with lymph node positivity identified during radical prostatectomy survived without further therapy [7].

Further supporting this notion, Seiler et al. showed that 57% of patients with a single affected lymph node detected during extended lymphadenectomy remained free of biochemical recurrence for up to 15 years without additional treatment [14].

Significantly more patients showed a positive qPCR than actually suffered a biochemical recurrence later on. Part of this difference can possibly be explained by healing as a result of surgical removal.

Indeed, a significantly higher sensitivity of the molecular method compared to conventional examinations has been demonstrated. Our study thus affirms earlier observations by Köllermann et al. and those from our own research group. However, the hypothesis investigated in this study that biochemical recurrence occurs earlier and more frequently in positive patients could not be confirmed. In this aspect, conventional histology has proven to be equivalent. Given the focus on metastasis, our average 5-year follow-up for this analysis is undoubtedly sufficient. Over this period, clinically relevant differences should have become apparent. Apparently, it is more likely that the detection of minimal metastases in distant lymph nodes does not automatically lead to further spread. This could be attributed to several reasons:

Firstly, it is possible that all affected areas were indeed surgically removed, leaving no further occult metastase within the patient. This is supported by the substantial sample size of an average of 24.7 lymph nodes. The German S3 guidelines for prostate cancer stipulate that at least 10 lymph nodes should be removed to provide a representative assessment of involvement. In our clinic, at the time, routine lymphadenectomy was consistently performed, which could be characterized as extended. Results from comparative prospective studies on whether extended lymphadenectomy (LAE) contributes to increased survival and/or merely results in more complications are currently unavailable. The SEAL-2 study is nearing the end of recruitment, with results expected in due course. Existing cohort comparisons have not demonstrated any benefit for patients treated with extended LAE [15].

Secondly, it is possible that micrometastases persisting in the remaining lymphatic tissue post-lymphadenectomy never translate into a clinical correlate. This could be attributed to the inherently slow growth of prostate cancer and/or the absence of as-yet-unknown signals that trigger further dissemination [16]. Factors such as altered diet or lifestyle may also play a role, as the experience of one's own cancer threat often leads to modified behavior [9].

Thirdly, it is possible that PSA RNA expression may not necessarily occur within viable malignant prostate tumor cells. Instead, it suggests the potential transfer of PSA RNA to lymph nodes via residual prostate cells. This phenomenon could occur after a prostate biopsy or other medical interventions.

Our decisive hypothesis, was that the presence of PSA expression in lymph nodes always indicates metastases [1]. However, other scientific studies have shown that PSA can originate from non-prostate cells and can therefore be detected in breast tissue, breast milk and female urine [17]. Contrary to this, numerous older pathological analyses indicate that while there are fundamentally PSA-negative prostate cells that somewhat reduce sensitivity, the substance is exclusively present in the prostate (100% specificity) [8]. The hypothesis of minimal baseline expression, detectable only by highly sensitive methods such as qPCR, raises the possibility of false-positive identification of micrometastases in lymph nodes after prostatectomy and warrants further investigation. We employed qPCR for detection. Naturally, RNA expression must precede actual protein production, so there should be no lower sensitivity than in the aforementioned pathological studies.

This theory is supported by the findings of several studies concerning the rationale for adjuvant radiation therapy [10]. In the past, it was routinely applied to many patients in attempt to combat a presumed occult tumor mass through a second therapeutic intervention. However, considering results indicating that adjuvant therapy provides no additional advantage in relation to progression survival compared to early salvage therapy, but is associated with significantly higher morbidity, such adjuvant therapy is no longer recommended as a routine practice today. Salvage radiotherapy should only be considered for those who actually experience biochemical recurrence.

Moreover, it is conceivable that the internationally recognized threshold for biochemical recurrence (multiple readings > 0.2 ng/ml or a single reading > 0.4 ng/ml) may be insufficiently sensitive for this inquiry. In a previous study conducted by our own group, albeit with a smaller patient cohort and a shorter follow-up period, but utilizing a substantially lower cut-off for defining biochemical recurrence, we observed a positive correlation between molecularly positive lymph nodes and recurrence [13].

It is possible that random distribution of tumor cells between the bisected lymph node halves could result in some cases where histology might have detected metastases if the other half had been examined. In cases of unequal tumor mass distribution, the results of further analyses in these specific cases could be inaccurate. However, we assume this affects only a very small number of samples.

Furthermore, in this context it is unproven that a certain mass of PSA-producing cells is required to initiate the release of the substance into the bloodstream or that a minimal vascular supply to lymphatic structures is necessary for the transfer of PSA into the blood.

These findings support the primary principle of “do no harm” outlined in the medical oath. We have demonstrated that increasing sensitivity with molecular biology techniques indeed reveals many more occult processes but also that this does not translate into clinical consequences. Therefore, there is no need for this method or any resulting intensified follow-up and/or additional therapy. Standard follow-up observations using conventional monitoring of PSA levels at regular intervals are sufficient for post-treatment surveillance.

So, the question raised in the title “More micrometastases, more recurrence?” must be answered negatively. These findings are in line with the latest evidence and patients can therefore avoid overdiagnosis and the associated overtreatment.

## Supplementary Information

Below is the link to the electronic supplementary material.

Supplementary file1 (DOCX 16 KB)

## Data Availability

No datasets were generated or analysed during the current study.
