# Supplementary material for: More micrometastases, more recurrence? The role of qPCR of PSA mRNA in lymph nodes during prostatectomy
Source: World J Urol. 2025 Jan 5;43(1):62. doi: 10.1007/s00345-024-05414-2 (PMC11700908; doi:10.1007/s00345-024-05414-2)
Supplement: Supplementary file 1 — Supplementary file1 (DOCX 16 KB) [file 345_2024_5414_MOESM1_ESM.docx]

Supplementary Figures:

|  |  | Histology | | total |
| --- | --- | --- | --- | --- |
|  |  | negative | positive |  |
| qPCR-PSA | negative | 58 | 3 | 61 |
|  | positive | 34 | 13 | 47 |
| total |  | 92 | 16 | 108 |

*Figure 4: qPCR vs Histology*

|  |  | Biochemical recurrence | | total |
| --- | --- | --- | --- | --- |
|  |  | negative | positive |  |
| Histology | negative | 61 | 29 | 90 |
|  | positive | 8 | 8 | 16 |
| total |  | 69 | 37 | 106 |

*Figure 5: Histology vs Recurrence.* This graph depicts the number of patients who were initially identified as positive in histology and later experienced biochemical recurrence.
*29 of 37 cases with positive tumor recurrence were not predicted by histology.* *This results in a sensitivity of 8/37 = 21.6%, specificity of 61/69 = 88.4%*, *PPV of 8/16 = 50.0% and NPV of 61/90 = 67.8%.*

|  |  | Biochemical recurrence | | total |
| --- | --- | --- | --- | --- |
|  |  | negative | positive |  |
| qPCR-PSA | negative | 41 | 18 | 59 |
|  | positive | 28 | 19 | 47 |
| total |  | 69 | 37 | 106 |

*Figure 6: qPCR vs Recurrence.* This graph depicts the number of patients who were initially identified as positive in qPCR and later experienced biochemical recurrence. 18 of 37 cases with positive tumor recurrence were not predicted by RTPCR. This results in a sensitivity of 19/37 = 51.4%, specificity of 41/69 = 59.4%, PPV of 19/47 = 40.4% and NPV of 41/59 = 69.5%.
